# Red-eared slider turtle*–Mycobacterium marinum* infection model

**DOI:** 10.1128/iai.00315-25

**Published:** 2025-10-07

**Authors:** Longlong Wang, Jijie Jiang, Mengke Hou, Zhe Wang

**Affiliations:** 1 Shanghai Key Laboratory of Veterinary Biotechnology, School of Agriculture and Biology, Shanghai Jiao Tong University 12474, Shanghai, China; 2 Shanghai Collaborative Innovation Center of Agri-Seeds, School of Agriculture and Biology, Shanghai Jiao Tong University 12474, Shanghai, China; Rutgers-New Jersey Medical School, Newark, New Jersey, USA

**Keywords:** *Mycobacterium*, *Mycobacterium marinum*, animal model

## Abstract

*Mycobacterium marinum* serves as an ideal model organism for studying tuberculosis due to its genetic similarity to *Mycobacterium tuberculosis*. However, there is a need for more suitable animal models to study *M. marinum* infections. In this study, we established a novel infection model using red-eared slider turtles (*Trachemys scripta elegans*). The turtles were infected with *M. marinum* via subcutaneous injection in the hind limb. Inoculation with >10^6^ CFU of *M. marinum* resulted in acute infection, causing mortality in at least 80% of turtles within five weeks, whereas 10^5^ CFU caused only 10% mortality. In subacute infections, *M. marinum* colonized and proliferated in various tissues for at least four weeks, with higher bacterial loads observed in the spleen and liver compared to the heart and lungs. Granuloma formation in the liver was correlated positively with bacterial load. Knockdown of adenylate kinase (ADK) in *M. marinum* reduced bacterial load by one order of magnitude in the liver and by half in the spleen, suggesting ADK as a potential drug target. Treatment with amikacin and moxifloxacin reduced bacterial load by approximately one order of magnitude in the liver and by half in the spleen. The red-eared slider turtle*–M. marinum* infection model developed in this study provides a robust tool for tuberculosis research.

## INTRODUCTION

Tuberculosis (TB), caused by *Mycobacterium tuberculosis* (*M. tuberculosis*), remains one of the most devastating infectious diseases worldwide, with an estimated 10 million new cases and 1.5 million deaths annually (1). Despite the availability of effective treatments, the emergence of multidrug-resistant (MDR) and extensively drug-resistant (XDR) strains of *M. tuberculosis* has posed significant challenges to global TB control efforts (2). Therefore, there is an urgent need to develop new therapeutic strategies and better understand the pathogenesis of TB.

*Mycobacterium marinum*, a close relative of *M. tuberculosis*, has been widely used as a model organism to study TB due to its genetic similarity and ability to infect a variety of hosts, including fish, amphibians, and mammals (3, 4). *M. marinum* shares approximately 99.4% 16S rRNA homology with *M. tuberculosis* and exhibits similar pathogenic mechanisms, such as the granuloma formation, which is characteristic of TB infections. However, the optimal growth temperature of *M. marinum* (30–32°C) limits its ability to cause systemic infections in mammals, making it necessary to develop alternative animal models that can better mimic human TB infections (5, 6).

Currently, zebrafish (*Danio rerio*) and mice are the most commonly used animal models for *M. marinum* infection studies. Zebrafish offer the advantage of transparency, allowing for real-time visualization of infection progression, but their small size limits the volume of inoculum and complicates drug delivery and tissue sampling (7). Mice, on the other hand, are widely used in TB research, but *M. marinum* infections in mice are typically restricted to localized infections in the tail or footpad due to the bacterium’s temperature sensitivity (8–10). These localized infections are prone to secondary infections from environmental microorganisms, which can interfere with accurate CFU counting and lesion measurement.

The red-eared slider turtle (*Trachemys scripta elegans*), a semi-aquatic reptile native to North America, has recently emerged as a promising model for studying how temperature determines reptiles’ sex (11). Red-eared slider turtles are natural hosts of *M. marinum* and can develop systemic infections that closely resemble human TB, including the formation of granulomas in internal organs, such as the liver and spleen. Additionally, the turtle’s ability to thrive at temperatures conducive to *M. marinum* growth (25–30°C) makes it an ideal host for studying the bacterium’s pathogenesis and evaluating potential therapeutic interventions.

In this study, we established a novel *M. marinum* infection model using red-eared slider turtles. We evaluated the effects of different inoculation doses on survival rates and bacterial load in various organs, and we investigated the role of adenylate kinase (ADK) in *M. marinum* pathogenesis using a CRISPRi-based knockdown approach (12). Furthermore, we assessed the efficacy of two second-line anti-TB drugs, amikacin (AMK) and moxifloxacin (MXF), in reducing bacterial load and granuloma formation in infected turtles. Our findings demonstrate that the red-eared slider turtle is a robust and versatile model for studying *M. marinum* infections, with potential applications in TB research and drug development.

## MATERIALS AND METHODS

### Animal

The red-eared slider turtles (shell length 9–10 cm, about 100 g weight) were purchased from a farm in Jinhua, China. All animal studies were conducted with the Regulations for the Administration of Affairs Concerning Experimental Animals, as mandated by the State Council of the People’s Republic of China.

### Bacteria and medium

*Mycobacterium marinum* M strain is a kind gift from Prof. Gao Qian of Fudan University. To construct the ADK knockdown strain, the sgRNA targeting *adk* was synthesized as forward and reverse primers (Forward:5′-GGGAGCGGTAGACCTTCATCCGGT-3′; Reverse: 5′-AAACACCGGATGAAGGTCTACCGC-3′). These primers were annealed and inserted into the *Esp*3I (Thermo Fisher)-digested pLJR962 vector (12). The recombinant plasmid was transformed into *M. marinum* by electroporation. ADK knockdown strain named as M.m-adk-sg416. *M. marinum* with empty pLJR962 vector was used as control strain. Wild-type, control, and knockdown strains were cultured in BSL2 at 30°C in standard Middlebrook 7H9 broth supplemented with 0.2% glycerol, 10% ADC, and 0.05% tyloxapol, or on 7H10 agar supplemented with 0.5% glycerol and 10% OADC. 20 µg/mL of kanamycin was added when necessary.

### Breeding condition and feeding

The turtles were kept in tanks and disinfected with potassium permanganate (KMnO_4_) solution after being received. The breeding room temperature was maintained at 25–30°C. High-quality turtle food containing 30% total protein was provided every two days.

Before infection, the turtles were acclimated to the breeding environment for at least twoweeks. To reduce the influence of other pathogens, 10 mg/kg of gentamycin was injected into the axilla of the forelimb for three days, followed by 10 mg/kg of levofloxacin for another three days. At least a three-day interval was necessary before infection. During the infection experiment, wastewater was treated with bleach at 1:10,000 for 3 hours before discharge.

### Infection experiment

For acute and subacute infection experiments, different concentrations of *M. marinum* (10^7^, 10^6^, and 10^5^ CFU) were resuspended in 50 µL saline and were injected subcutaneously in the hind limb. The turtles were dissected under sterile conditions once dead. At the end point of the infection experiment, the turtles were anesthetized with 7 mg/kg of Zoletil50, then euthanized by decapitation. After dissection, different organs were taken and homogenized for CFU counting. The tissue samples were also fixed using 4% paraformaldehyde for Hematoxylin and Eosin (H&E) staining or Acid-Fast Bacilli (AFB) staining.

For the tissue distribution experiment, 10^5^ *M. marinum* were resuspended in 50 µL of saline and injected subcutaneously in the hind limb. Five turtles were dissected weekly, and the spleen, liver, heart, and lung of each turtle were taken for CFU counting and histopathology examination.

### Gene knockdown and drug treatment

For gene knockdown, anhydrotetracycline (ATc) was injected intramuscularly into the forelimb axilla once daily at 200 µg/kg. Turtles were euthanized at day 7, and the organs were subjected to CFU counting and histopathology examination.

For drug treatment, at seven days post-infection, AMK or MXF was injected intramuscularly into the forelimb axilla once daily at 5 mg/kg. Turtles were euthanized at 21 days post-infection for CFU counting and histopathology examination.

## RESULTS

### *M. marinum* acute infection causes turtles' mortality

**Fig 1:**
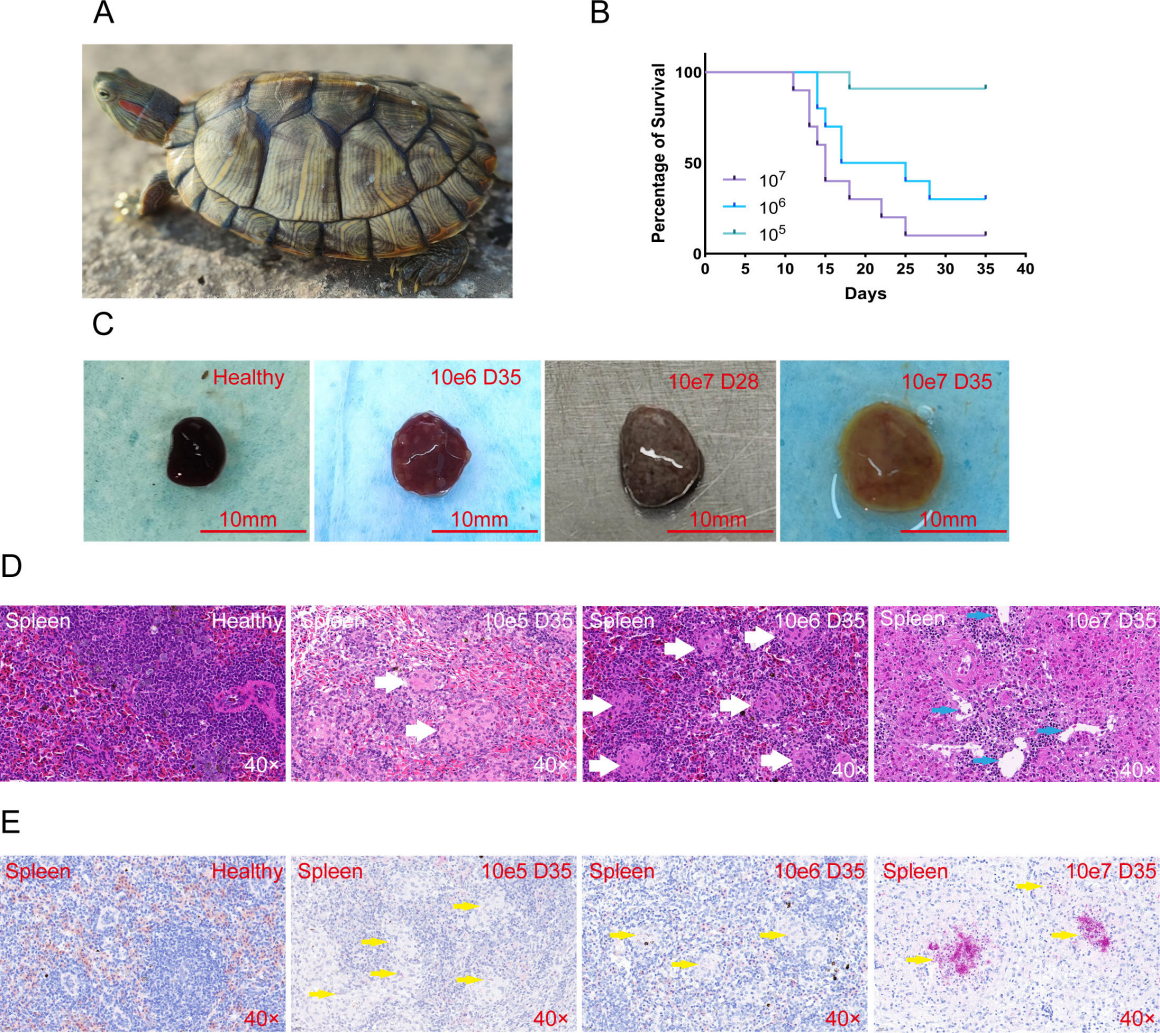
*M. marinum* acute infection causes turtles’ death and spleen lesions. (**A**) Red-eared slider turtles (*Trachemys scripta elegans*)*.* (**B**) The survival curve of turtles infected with 10^7^, 10,^6^ or 10^5^ CFU *M*. *marinum*. Long-term infection with *M. marinum* causes macroscopic lesions in the appearance of the spleen (**C**) and the formation of granulomas (**D**). Non-necrotic granulomas are marked with white arrows. Necrotic granulomas are marked with blue arrows. (**E**) Acid-fast bacilli staining of spleen samples. AFB-positive *M. marinum* (red-colored) are marked with yellow arrows.

A total of 30 red-eared slider turtles (Fig. 1A) were randomly divided into three groups, and each group was injected with different concentrations of *M. marinum* (10^7^, 10^6^, and 10^5^ CFU, *n* = 10). The first mortality occurred in the 10^7^ group at 10 days post-infection (dpi). At the end point of this experiment (35 dpi), 10^6^ CFU inoculation caused 80% mortality (8/10), while 10^7^ CFU inoculation caused 90% mortality (9/10). Only 10% mortality (1/10) was observed in the 10^5^ group (Fig. 1B). We dissected turtles that survived until 35 dpi. The spleens from 10^7^ group showed significant enlargement, with a color changed to orange compared to the brownish-red of healthy spleens (Fig. 1C). Multiple white lesions also appeared on the spleen surface from the 10^6^ group (Fig. 1C). No significant changes were observed in the spleens from the 10^5^ group. According to histopathology results, long-term infection with *M. marinum* resulted in the development of granulomas in the spleen. At 10^5^ and 10^6^ groups, *M. marinum* infection caused non-necrotic granuloma formation (white arrows), which has well-circumscribed aggregates of epithelioid histiocytes and multinucleated giant cells. There is no central necrosis, and the granulomas appear uniform and cohesive (Fig. 1D). The number of non-necrotic granulomas was calculated, with 10–15 per square millimeter in the 10^6^ group and 5–10 per square millimeter in the 10^5^ group. At 10^7^ group, lymphocytes were drastically reduced, and non-necrotic granulomas filled the entire tissue (Fig. 1D). High bacteria load also caused typical necrotic granuloma formation, which has cavitary lesions encircling macrophages (blue arrows). The number of necrotic granulomas in the spleen was calculated, with approximately five cavitary lesions per square millimeter. Numerous non-necrotic granulomas were observed but were difficult to quantify due to their dense and indistinct boundaries. With AFB staining, *M. marinum* (red-colored) was present in the center of granulomas (Fig. 1E). In the 10^5^ and 10^6^ groups, the bacteria were observed as solitary or small groups, predominantly in epithelioid macrophages, consistent with non-necrotic granuloma formation in these groups. By contrast, extensive, classical cording-like bacteria were observed in the 10^7^ group, and this morphology is consistent with extracellular bacteria contained inside the necrotic core of the granulomas.

### *M. marinum* colonizes and replicates in various organs

**Fig 2:**
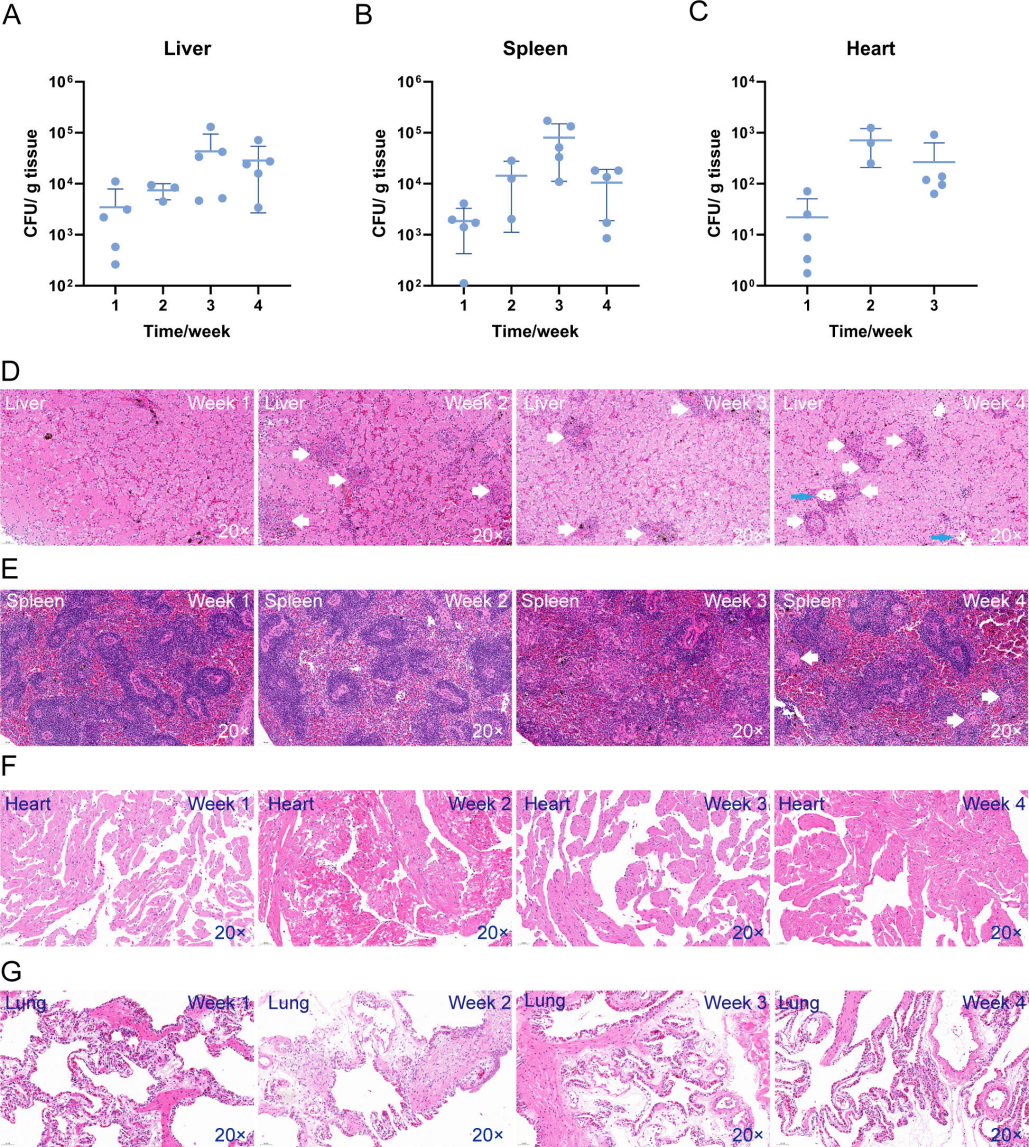
*M. marinum* colonizes and replicates in different organs. Each turtle was infected with 10^5^ *M. marinum* by subcutaneous injection of the hind limb. The bacteria load of the liver (**A**), spleen (**B**), and heart (**C**). *M. marinum* infection caused granuloma formations in the liver (**D**) and spleen (**E**), but not in the heart (**F**) or lung (**G**). Non-necrotic granulomas: white arrows. Necrotic granulomas: blue arrows. Error bars represent mean ± standard deviation (SD). Downward-pointing bars are not shown when the mean − SD results in a negative value.

To investigate the distribution of *M. marinum* in different tissues post-infection, a total of 20 turtles were injected with 10^5^ bacteria. Five turtles were sacrificed and dissected every week until the fourth week. Liver, spleen, heart, and lung were taken for CFU counting. As shown in Fig. 2, *M. marinum* colonized and replicated in liver, spleen, and heart. From the first to the third week, the bacterial load in the liver (Fig. 2A) and spleen (Fig. 2B) gradually increased up to 10^5^ CFU per gram tissue and began to decrease in the fourth week. According to histopathology results, non-necrotic granulomas were first observed in the liver at the second week (Fig. 2D) and in the spleen at the fourth week (Fig. 2E).

Compared to the spleen and liver, the heart exhibited a lower bacterial load (Fig. 2C) during the initial three weeks, and no bacteria were detected at the fourth week. The lungs were also taken for CFU counting, but only a few colonies grew on the minimal dilution plates. We also observed more environmental bacteria in the lung samples, which hindered CFU counting. Inconsistent with the low bacterial load, there was no clear non-necrotic granuloma or inflammatory infiltration in the lung or heart (Fig. 2F and G).

### ADK knockdown impairs *M. marinum* colonization and replication *in vivo*

**Fig 3:**
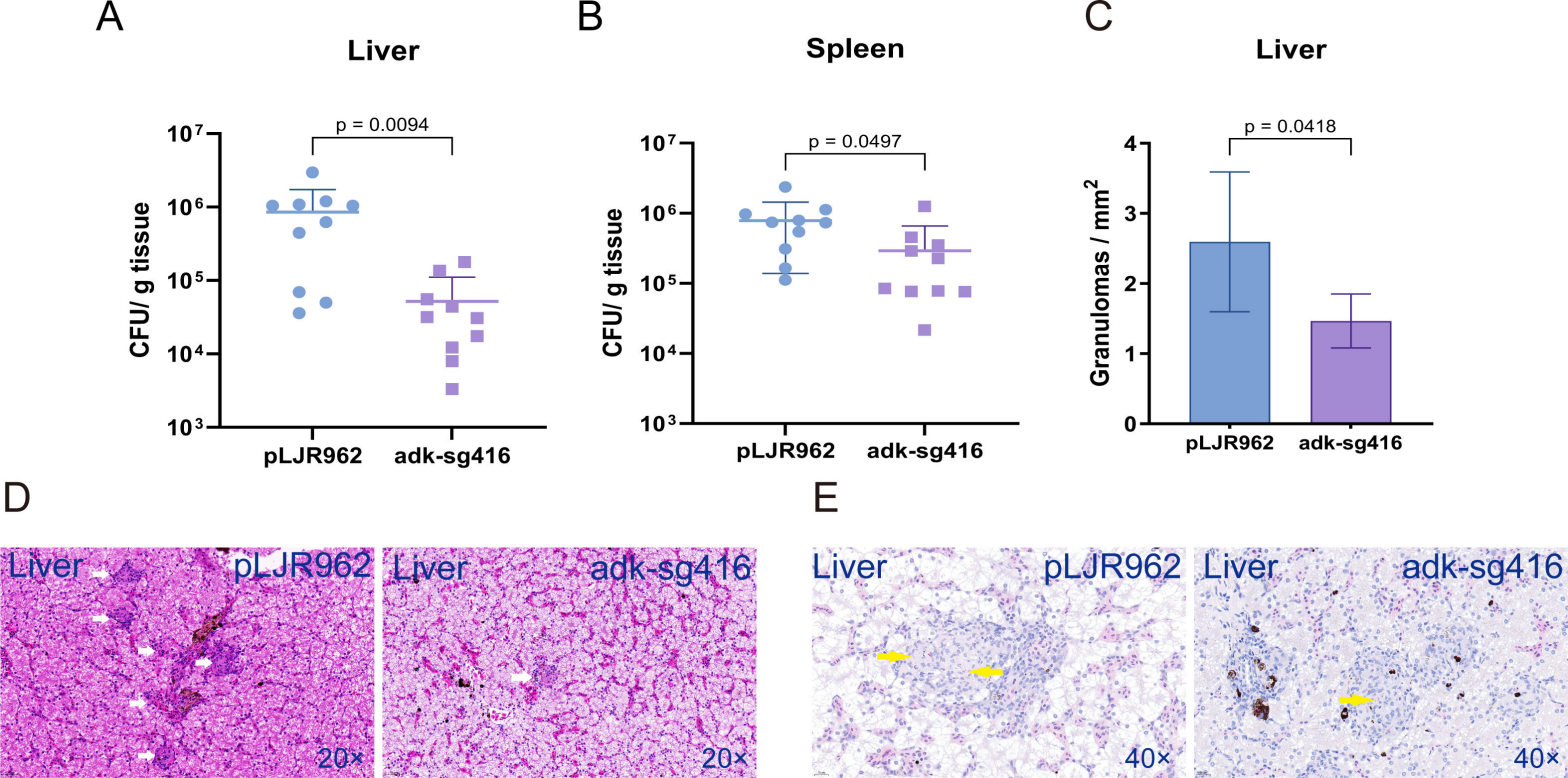
Knockdown of ADK impaired colonization and replication of *M. marinum in vivo*. ADK knockdown with ATc for one week reduced bacteria load in liver (**A**) and spleen (**B**) as well as the formation of granulomas in liver (**C and D**). AFB-positive *M. marinum* was observed in the center of granulomas (**E**). Non-necrotic granulomas: white arrows. AFB-positive *M. marinum*: yellow arrows. A two-tailed unpaired *t*-test was used to analyze the significance of data differences. Error bars represent mean ± SD. Downward-pointing bars were not shown when mean − SD results in a negative value.

The adenylate kinases of *Mycobacterium tuberculosis* have been supposed to be a promising drug target (13, 14). Using CRISPRi technology, an anhydrotetracycline (ATc)-induced *adk* knockdown strain was constructed and named as adk-sg416. The strain carrying the pLJR962 vector was used as a negative control. After ATc treatment for one week, the bacteria load reduced one order of magnitude in liver (Fig. 3A) and half in spleen (Fig. 3B). Histopathological analysis showed that the pLJR962 group formed more granulomas in the liver than the *adk* knockdown group (Fig. 3C and D). AFB-positive *M. marinum* was observed in the center of non-necrotic granulomas (Fig. 3E).

### Amikacin and moxifloxacin reduce *M. marinum* load *in vivo*

**Fig 4:**
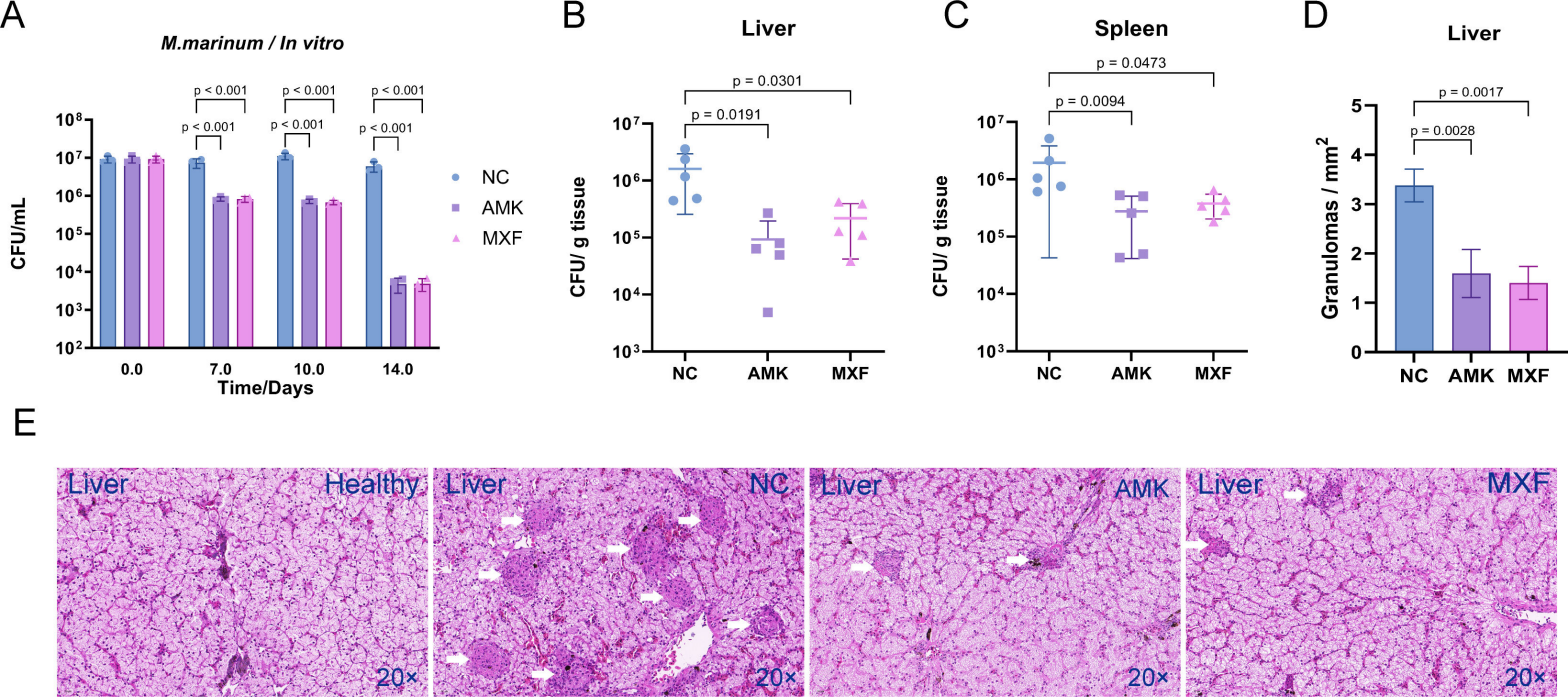
Amikacin and moxifloxacin killed *M. marinum in vivo*. (**A**) Amikacin and moxifloxacin showed good bactericidal activity against *M. marinus in vitro*. Amikacin and moxifloxacin treatment for two weeks reduced bacteria load in the liver (**B**) and spleen (**C**) as well as the formation of granulomas in liver (**D and E**). Non-necrotic granulomas: white arrows. Dunnett’s multiple comparisons test was used in conjunction with one-way ANOVA to generate the individual *P* values. The error bars represent mean ± SD. Downward-pointing bars were not shown when mean − SD results in a negative value.

Evaluating the efficacy of drugs in animal models is an important procedure of the anti-tuberculosis drug development. In this research, we first determined the minimum inhibitory concentration (MIC) of amikacin (1 µg/mL) and moxifloxacin (0.5 µg/mL) using the Alamar Blue staining method. In 7H9 + ADC medium, AMK and MXF had the same bactericidal activity at 5 ug/mL, and approximately 90% bacteria were killed at day 7 and 99.9% at day 14 (Fig. 4A). In the turtle model, 15 turtles were randomly divided into three groups and inoculated with 5 × 10^5^ CFU of *M. marinum*. After one week, 5 mg/kg AMK or MXF was injected intramuscularly once daily for 14 days. Both drugs reduced bacterial load by approximately one order of magnitude in the liver (Fig. 4B) and by half in the spleen (Fig. 4C). Drug treatment also reduced granuloma formation in the liver compared to the negative control group (Fig. 4D and E).

## DISCUSSION

For *M. marinum*, zebrafish and mouse are usually selected to construct infection model (15). However, the small size of zebrafish makes injection, drug administration, and dissection difficult. Although some researchers alternatively used goldfish (16), the quality of culture water is not easy to control without a recycling system. As reptiles, red-eared slider turtles breathe with lungs, reducing their dependence on water quality compared to aquatic species. The turtles’ size (9–10 cm) facilitated procedures including breeding, drug administration, and dissection. The organ size of turtles allowed the formation of sufficient granulomas to assess the tissue damage by counting the number of granulomas. The granulomas formed in the liver and spleen of turtles post-*M. marinum* infection were similar to those in tuberculosis (6). However, similar granulomas were not observed in turtle lungs, possibly due to differences in histotropism between *M. marinum* and *M. tuberculosis*.

The *M. marinum* has an optimum growth temperature at 30–32℃ (8), which means the bacteria cannot colonize and replicate normally in the internal organs of mammals; thus, the local infection in mouse tails and footpads is chosen by researchers (9, 10). In these models, the tail and footpad are in constant contact with bedding material and feces, and the secondary infection caused by environmental microorganisms posed a significant hindrance to the measurement of visible tail lesions and CFU counting. With antibiotic pretreatment before infection, along with physical barriers and the immune system, the internal organs of turtles have a low chance of being infected with other pathogens. According to our previous observation, there was no swelling and suppuration at the inoculation site post-infection. This reduces the risk of secondary infection by environmental microorganisms.

As a pathogen causing systemic infection, the inoculation sites of *M. marinum* are not restricted to the hind limbs; other sites, such as neck, forelimbs, armpits, and tail, are acceptable. We do not recommend the head because the thin skin of the head may not be able to bear the inoculation volume. Neck and tail intravenous injection methods are also worth trying. Some clinical veterinarians prefer to use this drug delivery method to treat high-value pet turtles.

Moxifloxacin is a fourth-generation fluoroquinolone antibiotic (17). Amikacin is a semisynthetic aminoglycoside antibiotic (18). These two drugs are among the most important second-line drugs for multidrug-resistant tuberculosis. In this study, MXF and AMK were selected because they have good bactericidal activity against *M. marinum in vitro* and good water solubility. Thus, we administered the drugs by intramuscular injection. Of course, other delivery methods, such as intravenous and caudal administration and subcutaneous injection, are also possible. In our previous study, we found that rifampicin, rifapentine, and rifabutin also possessed potent bactericidal activities (data not shown) *in vitro*; however, due to their poor solubility in aqueous solution, they cannot be administered via intramuscular injection. Red-eared slider turtles have a strong appetite and can be kept dry for a short time. Next, we will try to give drugs by mixed feed, which is meaningful for oral drugs.

As a widely popular pet species, the red-eared slider turtle is abundantly available in the market, rendering it both cost-effective and easily accessible. Its robust feeding behavior, strong disease resistance, and high environmental adaptability make it an exceptionally manageable model organism for laboratory studies (19). Notably, other turtle species commonly found in the pet trade, such as the common slider turtle (*Trachemys scripta scripta*), hybrids between red-eared and common slider turtles, and the Chinese pond turtle (*Mauremys reevesii*), exhibit similar advantageous traits, suggesting their potential utility in establishing *M. marinum* infection models (20, 21). Furthermore, it would be of significant interest to investigate whether *M. tuberculosis* and other pathogenic non-tuberculous mycobacteria (NTM) can also establish infection models in these turtle species, which could broaden the scope of their application in mycobacterial research.

In conclusion, we have constructed a novel animal infection model for *M. marinum*. This turtle*–M. marinum* infection model can be used for functional study of *Mycobacterium* genes, evaluation of anti-tuberculosis drugs, as well as novel drugs and vaccines development.

## SUPPLEMENTAL MATERIAL

Supplemental material iai.00315-25-s0001.docx Guidelines for feeding and dissection of the red-eared slider turtle.

ASM does not own the copyrights to Supplemental Material that may be linked to, or accessed through, an article. The authors have granted ASM a non-exclusive, world-wide license to publish the Supplemental Material files. Please contact the corresponding author directly for reuse.
