# Supplementary material for: Red-eared slider turtle–Mycobacterium marinum infection model
Source: Infect Immun. 2025 Oct 7;93(11):e00315-25. doi: 10.1128/iai.00315-25 (PMC12604484; doi:10.1128/iai.00315-25)
Supplement: Supplemental material — Guidelines for feeding and dissection of the red-eared slider turtle. [file iai.00315-25-s0001.docx]

### Guidelines for Feeding and Dissection of the Red-Eared Slider Turtle

### The red-eared slider turtle, a popular pet due to its strong environmental adaptability, disease resistance, and robust appetite, is relatively easy to care for. However, feeding recommendations for researchers are provided here. Additionally, the red-eared slider turtle possesses unique physiological structures as a reptile. Based on extensive experimental procedures, we offer dissection guidelines to assist researchers in obtaining organ tissues accurately and facilitating smooth experimental progress.

### Rearing Guidelines

### Turtle Selection: We recommend purchasing turtles with a carapace length of 8-10 cm. Smaller turtles may be difficult to handle, while larger turtles, due to their advanced carapace calcification, can complicate dissection procedures.

### Source: Whenever possible, procure turtles from local or nearby farms. Long-distance transportation may influence the turtles' health.

### Acclimatization: Upon arrival, do not immediately place the turtles in water. Allow them to acclimate to the rearing environment's temperature for two hours. Then, place them in water with a depth just above the carapace. During this time, the turtles will drink and defecate. After one hour, discard the water and replace it with clean water for rearing.

### Disinfection: During the first two days, disinfect the turtles' skin using a potassium permanganate solution (Figure 1). The concentration of the solution should be inversely proportional to the soaking duration; we recommend a low concentration with extended soaking time.


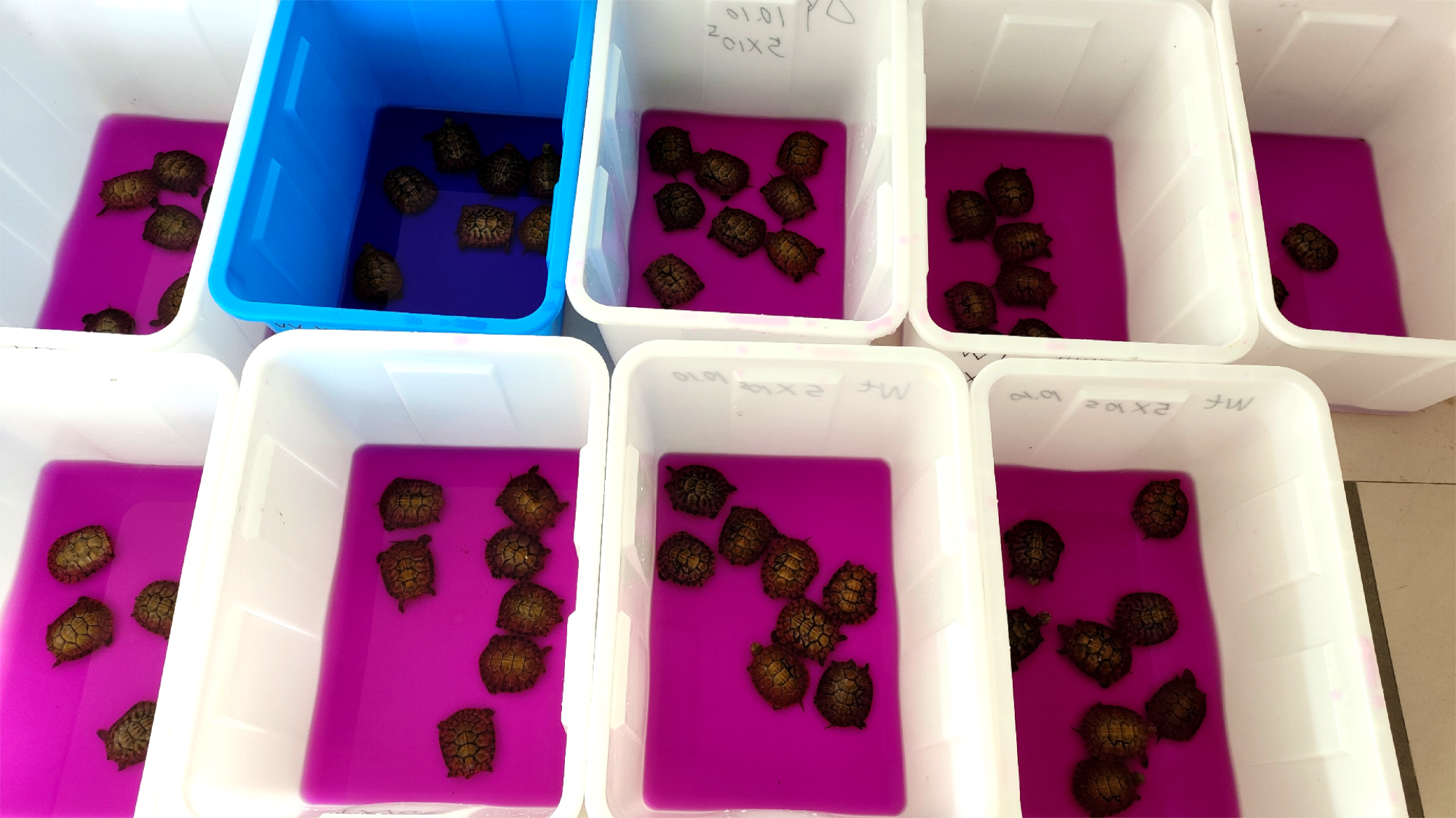


Figure 1. Disinfect the turtles' skin using a potassium permanganate solution

### Space: Ensure each turtle has adequate space for movement.

### Water Quality: Use fresh, aerated water (aerated for over 24 hours) for rearing, and maintain a water temperature consistent with the rearing environment (Figure 2).


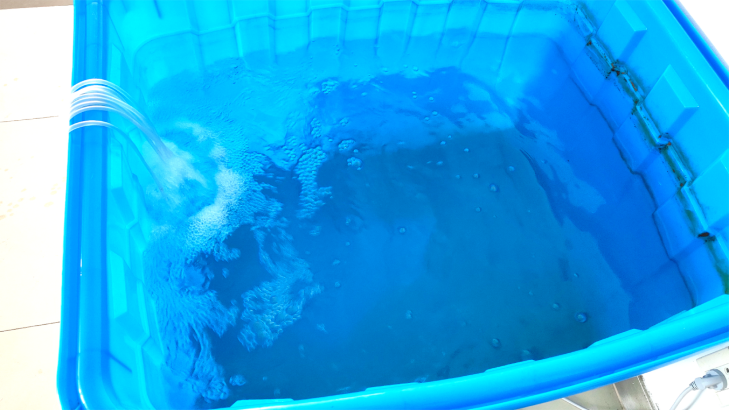


Figure 2. Feeding water needs to be aerated for more than 24 hours

### Feeding: Provide high-quality turtle feed with a protein content exceeding 30% (Figure 3). Feed the turtles once every two days, offering an amount they can consume within 30 minutes. Replace the water before and after feeding to maintain hygiene and ensure clean living conditions.


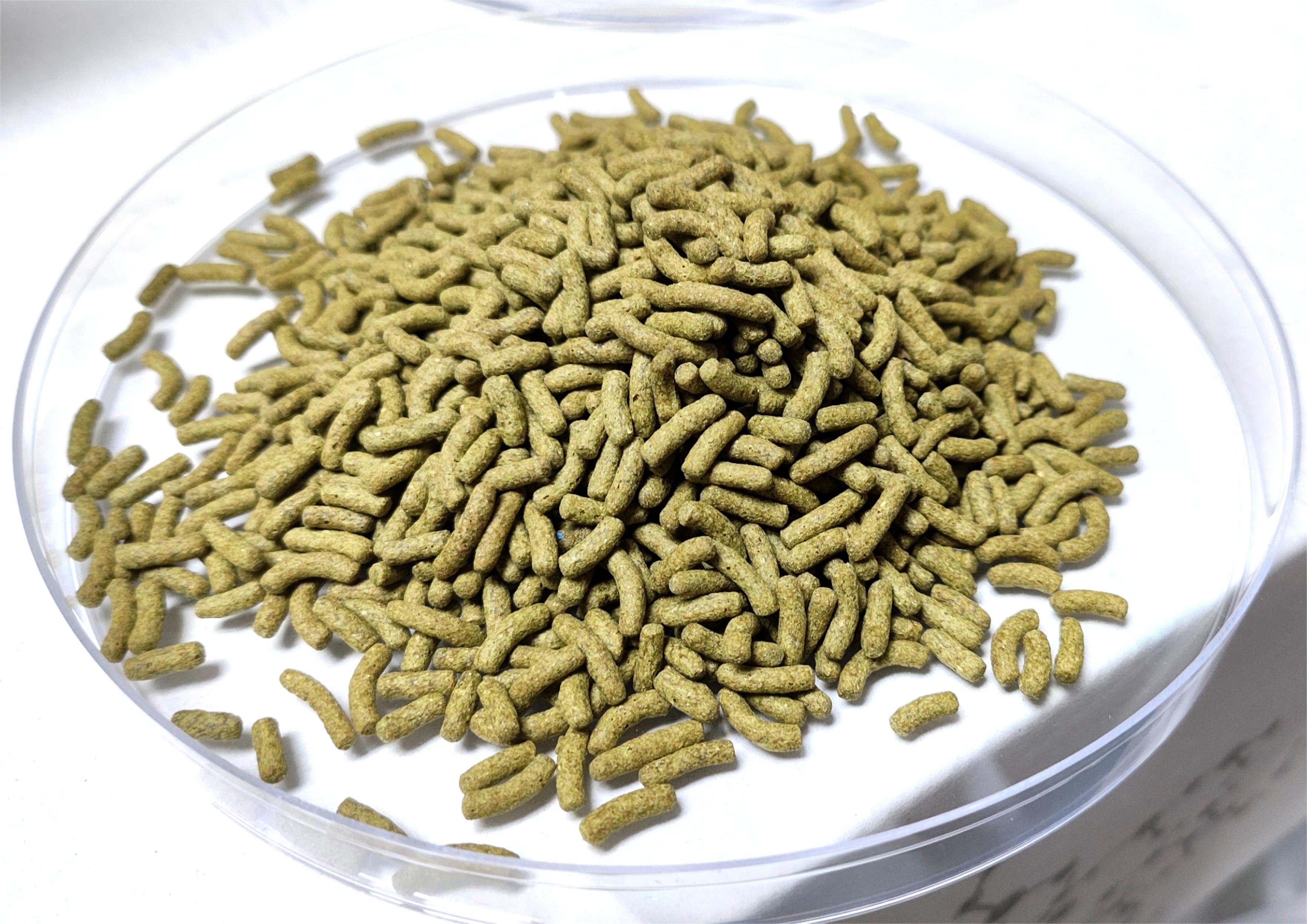


Figure 3. High-quality turtle feed with a protein content exceeding 30%.

### Wastewater Treatment: During infection phases, sterilize the rearing wastewater. We recommend using chlorine-based disinfectants or potassium permanganate solutions for effective wastewater disinfection.

### Infection and Drug Administration


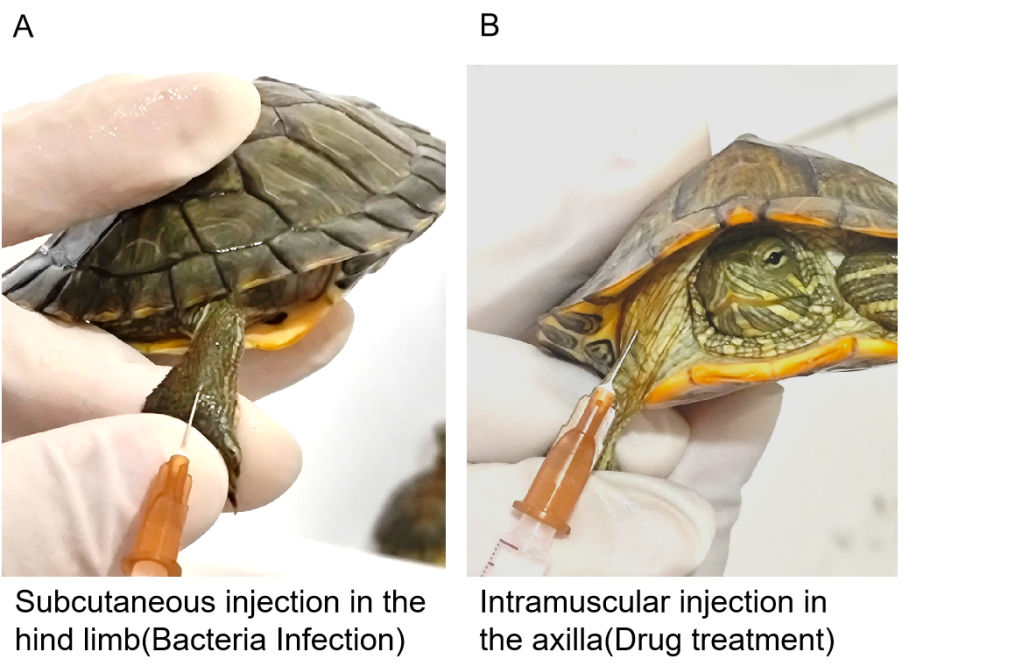


Figure 4. Subcutaneous injection in the hind limb and intramuscular injection in the armpit area

**Infection:** We recommend subcutaneous injection in the hind limb (Figure 4A).

**Drug Administration:** We recommend intramuscular injection in the armpit area (Figure 4B). Avoid areas near the neck, this can be injected into the trachea or arteries.

**Anesthesia and Dissection Protocol**


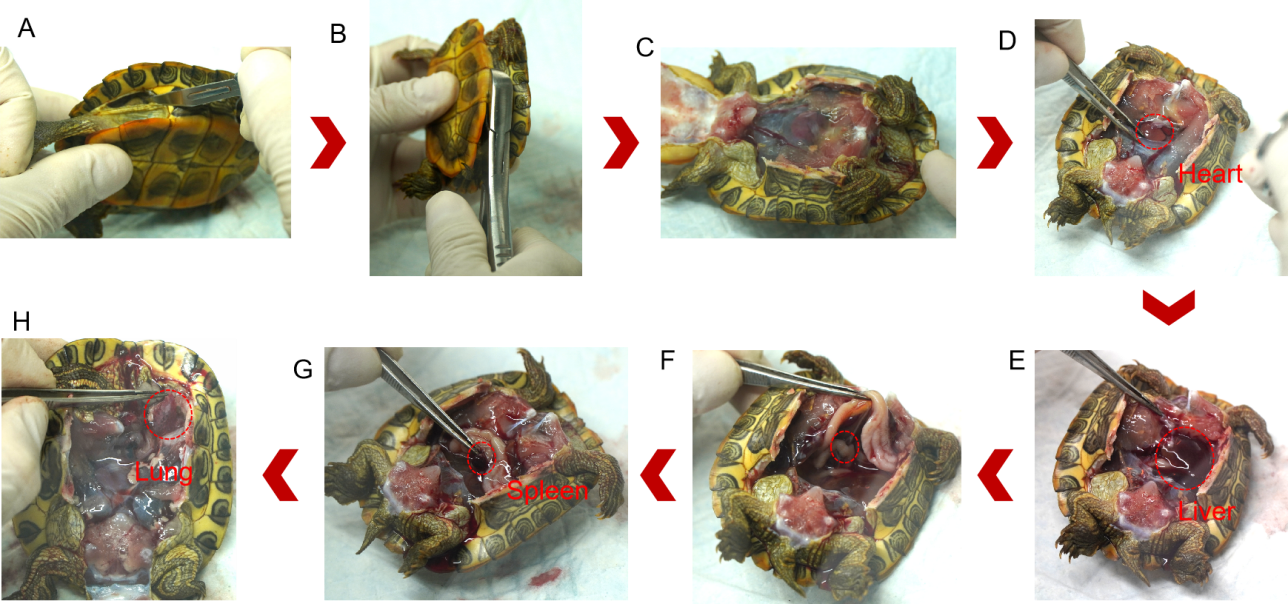
Figure 5. Anatomy of red-eared slider turtle

**Anesthesia:** Weigh each turtle and intramuscular injection of Zoletil 50 at a dosage of 7 mg/kg in the armpit area for anesthesia (Figure 4B). After 5-10 minutes, euthanize the deeply anesthetized turtle by decapitation.

### Initial Incisions: Use a scalpel to cut the skin connections between the limbs, head, tail, and the plastron (ventral shell) (Figure 5A).

### Carapace and Plastron Separation: Using orthopedic surgical scissors, carefully cut through the connective tissue joining the carapace (dorsal shell) and plastron on both sides (Figure 5B).

### Plastron Removal: With a scalpel, dissect the connective tissue between the plastron and the thoracic-abdominal cavity. Lift and remove the plastron to expose the internal organs (Figure 5C).

### Heart Extraction: Carefully cut through the mesentery of the abdominal cavity to reveal the thoracic-abdominal cavity. The heart is located centrally within this cavity (Figure 5D).

### Liver Extraction: The liver is situated directly below the heart and is relatively large. For sampling, excise a small portion from the left lobe (Figure 5E).

### Spleen Extraction: Lift the liver and stomach to expose the underlying structures. The spleen is encased within the connective tissue posterior to the intestines. Carefully free this section of the intestines to access and remove the spleen (Figure 5FG).

### Lung Extraction: The lungs are located in the dorsal region of the thoracic-abdominal cavity, closely adhered to the carapace (Figure 5H). After the turtle's death, the lungs will contract sharply.

Note: There is a large bladder in the abdominal cavity that stores urine. Punctures were avoided during dissection.
